# Levels of circulating angiotensin‐converting enzyme 2 are affected by acute exercise and correlate with markers of physical fitness in male athletes

**DOI:** 10.14814/phy2.16161

**Published:** 2024-07-17

**Authors:** Stefan M. Reitzner, Eric B. Emanuelsson, Carl Johan Sundberg

**Affiliations:** ^1^ Department of Physiology and Pharmacology Karolinska Institutet Stockholm Sweden; ^2^ Department of Women's and Children's Health Karolinska Institutet Stockholm Sweden; ^3^ Department of Learning, Informatics, Management and Ethics Karolinska Institutet Stockholm Sweden; ^4^ Department of Laboratory Medicine Karolinska Institutet Huddinge Sweden

**Keywords:** ACE2, acute exercise, ELISA, high‐level athletes, pre‐COVID, untrained

## Abstract

While under physiological conditions angiotensin‐converting enzyme 2 (ACE2) is an antagonist of vasoconstrictive agents in the renin–angiotensin–aldosterone system (RAAS), in the context of SARS coronavirus 2 (SARS‐CoV‐2) ACE2 serves as the gateway into cells. Furthermore, RAAS has previously been shown to be influenced by exercise training and is suggested to be involved in skeletal muscle mass maintenance. Given this connection, the investigation of circulating ACE2 plasma protein concentration before and following acute and chronic endurance and resistance exercise could increase the understanding of the implications of the exposure of athletes to SARS‐CoV‐2. Therefore, this study investigated levels of circulating ACE2 in lifelong high‐level trained endurance and resistance athletes and control subjects in response to either acute endurance or resistance exercise. Results show no baseline differences in absolute ACE2 concentration between groups, but a strong negative correlation with levels of fitness and positive correlation with BMI in control subjects. Furthermore, acute endurance exercise significantly increased ACE2 levels across all groups, but only in the strength group in response to resistance exercise. This indicates that circulating ACE2 plasma levels are influenced by levels of fitness and health, and that acute endurance exercise has a stronger effect on plasma ACE2 levels than resistance exercise.

## 1 INTRODUCTION

Under physiological conditions, the angiotensin‐converting enzyme 2 (ACE2) is part of the renin–angiotensin–aldosterone system (RAAS) that regulates amongst others blood pressure and vascular resistance. In that system, ACE2 is responsible for the cleavage of the vasoconstrictive angiotensin II into vasodilating angiotensin 1–7 (Bhalla et al., 2020). As such, it is a contributing antagonist to angiotensin‐converting enzyme (ACE) which generally results in an increase in blood pressure by constriction of blood vessels and increased renal sodium and water reabsorption. However, following the 2003–2005 outbreak of the severe acute respiratory syndrome (SARS) epidemic, membrane‐bound ACE2 was first identified as the entry point of the SARS coronavirus (SARS‐CoV) into cells (Kuba et al., 2005), and subsequently showed to also be the access point into cells for SARS‐CoV‐2, which caused the COVID‐19 pandemic (Hoffmann et al., 2020; Jackson et al., 2022). A recent review has furthermore highlighted the implication of ACE2 and angiotensin 1–7 in the maintenance of skeletal muscle mass and health and a potential ACE2‐associated path for SARS‐CoV‐2 invasion of skeletal muscle, subsequently contributing to aggravated, system‐wide inflammation (Yamamoto et al., 2020). In a large study of persons infected with SARS‐CoV‐2 it was shown that those that before the COVID‐19 pandemic reported high physical activity levels had less than half the risk of severe COVID‐19 disease compared to individuals that were consistently inactive (Sallis et al., 2021). However, no study to date investigated a potential correlation between physical activity background and plasma levels of ACE2. In addition to the potential links between exercise and COVID‐19 via ACE2, blood pressure and skeletal muscle, recent years have seen an ongoing debate about the influence and benefits of physical activity on the immune system as a whole (Nieman & Wentz, 2019). Some results suggest that the intensity of the exercise performed is important as highlighted in another recent review by Campbell et al. (Campbell & Turner, 2018). Another recent publication indicates that baseline levels of circulating ACE2 can predict disease progression and outcome in SARS‐CoV‐2 infected patients (Kragstrup et al., 2021), which might have interesting implications for athlete SARS‐CoV‐2 pathogenesis. Furthermore, blood ACE2 levels have been shown to be higher in patients already infected with SARS‐CoV‐2 than in healthy control subjects (Lundström et al., 2021).

Despite this new evidence of the high relevance of ACE2 in the pathophysiology of COVID‐19 and the existence of a potential mechanism of the involvement of exercise in ACE2‐related regulation, the effect of acute and lifelong exercise training on ACE2 levels in circulating blood has not been investigated to date. Thus, the aims of this study were to investigate ACE2 plasma levels at resting baseline and following acute endurance and resistance exercise in healthy, lifelong high‐level endurance and resistance athletes and non‐exercising control subjects collected before the appearance of SARS‐CoV‐2.

## 2 METHODS

This study was reviewed and approved by the Stockholm regional ethics board (Dnr: 2016/590‐31) under observance of the declaration of Helsinki. Twenty‐four healthy, nonsmoking men aged 33–52 have provided written consent to participate in this study and have previously been recruited after filling in a questionnaire to assess their training history and health status, excluding individuals regularly on medication, with respiratory or lung problems, relevant hospital visits during the last 5 years, and a number of common conditions such as high blood pressure, metabolic, blood and heart disorders, or musculoskeletal diseases. Furthermore, individuals that had hormone‐based therapies as well as smokers were excluded. To additionally assess physical capacity, a screening process including peak oxygen uptake test (V̇O_2_peak test) maximum knee torque test was used after which all suitable subjects were fit into one of three groups: resistance trained (SG), endurance trained (EG), and control (CG). V̇O_2_peak was performed on a stationary bike and initial resistance individually set depending on expected subject performance. Following 5 min of warm‐up, resistance was increased incrementally between 16.6 and 26.6 W·min^−1^ until exhaustion. All subjects reached a respiratory exchange ratio (RER) above 1.06. Leg strength was measured by unilateral isokinetic knee torque test using Biodex. Following familiarization and 5 min of warm‐up on a stationary bike, each subject performed three maximum effort attempts, with the highest valid attempt being counted. Resistance and endurance trained athletes were required to have either a resistance‐ or an endurance‐based training history at a high level for at least 15 years, respectively. The groups were by design clearly separated by both VO_2_peak and maximum knee torque. For more details about inclusion and group separation see Reitzner et al. (2024). All subjects were naïve to SARS‐CoV‐2 as the collection was completed before the outbreak of the pandemic. All subjects completed both a single bout of endurance (EE) and resistance (RE) exercise, which were performed in a randomized order with at least 1 month of washout in between, and food intake was controlled for by a standardized breakfast. Subjects were pushed to their maximum performance in each session, resistance exercise goals consisted of nine sets of eight repetitions at 80% of their measured one repetition maximum, endurance exercise goals consisted of 30 min of cycling at 75% of their individual *W*_peak_. Peripheral venous blood was taken from *Fossa cubitalis* before and directly after and at 1 h and 3 h after one single bout of exercise using EDTA as anticoagulant. Blood plasma was isolated in two subsequent centrifugations at 3000*g* for 10 min and analyzed using the Adipogen ACE2 ELISA kit (#AG‐45B‐0023‐KI03). Statistics were calculated by ANOVA using the Tukey post hoc test to compare groups at baseline, subject characteristics and the post timepoint to pre in the acute exercise response, correlation analysis using Pearson's correlation, and multiple linear regression and interaction analysis in R 3.6.0.

## 3 RESULTS

Characteristics of the included subjects are shown in Table 1. The three groups were clearly separated by both measures of physical fitness, VO_2_peak and maximum knee torque. Furthermore, athletes of SG had a higher body weight and BMI compared to CG and EG (Table 1).

**TABLE 1:** Subject group baseline characteristics.

Characteristics	CG	EG	SG
Age (years)	44 ± 6	42 ± 5	39 ± 6
Height (cm)	181 ± 6	181 ± 4	180 ± 5
Weight (kg)	76 ± 8	73 ± 4	92 ± 12*, ^§^
BMI (kg⋅cm^−2^)	23.2 ± 2.4	22.3 ± 0.5	28.3 ± 2.9*, ^§^
Peak torque (Nm)	180.6 ± 30.8	198.3 ± 25.5	286.43 ± 28.1*, ^§^
VO_2_peak (mL⋅kg BW^−1^⋅min^−1^)	36.2 ± 4.4	69.0 ± 7.2*	40.21 ± 6.9^§^

*Note*: Mean ± SD.

* *p* > 0.05 compared to control.

^§^ *p* > 0.05 compared to endurance.

**FIGURE 1:**
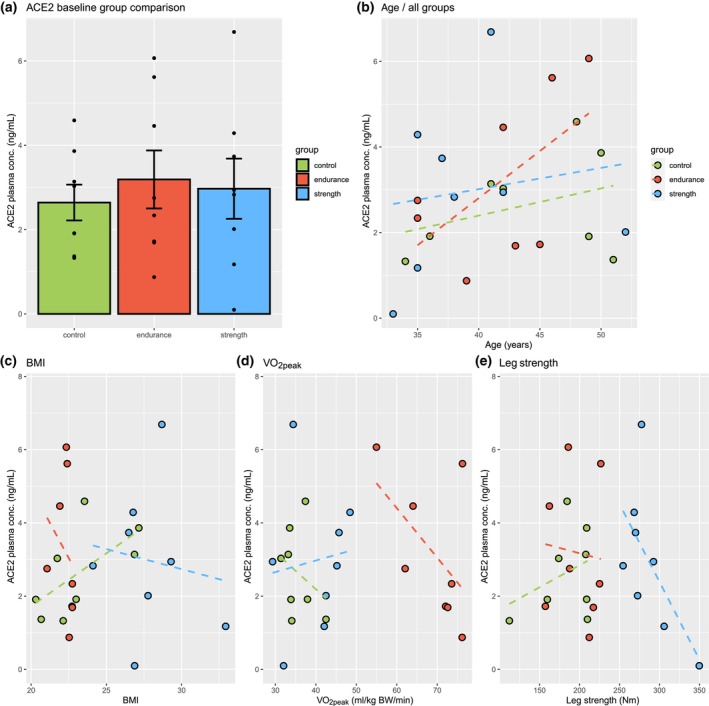
Baseline characteristics and correlations of control (CG), endurance (EG), and strength group (SG). (a) Baseline ACE2 plasma levels by group. (b) Correlation of age and baseline ACE2 plasma levels separated by group. (c) Correlation of BMI and baseline ACE2 (CG: *R* = 0.63, EG: *R* = −0.23, and SG: *R* = −0.14). (d) Correlation of VO2max and baseline ACE2 plasma levels (*R* = −0.3, −0.54, and 0.11, respectively). (e) Correlation of leg strength and baseline ACE2 plasma levels (*R* = 0.35, −0.08, and − 0.63, respectively).

Plasma levels of circulating ACE2 at baseline ranged from 0.22 ng/mL to 7.62 ng/mL, with CG, EG, and SG averages being 2.64 ng/mL (±0.42), 3.19 ng/mL (±0.69), and 2.97 ng/mL (±0.71) respectively with no significant differences between groups (Figure 1a). At baseline, ACE2 plasma levels in CG were positively and strongly correlated with BMI (*R* = 0.63, Figure 1c), and in EG and SG there was a strong negative correlation with their respective markers of physical fitness, VO_2_peak (EG; *R* = −0.54, Figure 1d), and peak torque (SG; *R* = −0.63, Figure 1e). Circulating ACE2 plasma levels correlation with age is shown in Figure 1b and was strong in EG (*R* = 0.58), moderate in CG (*R* = 0.35), and weak in SG (*R* = 0.15).

**FIGURE 2:**
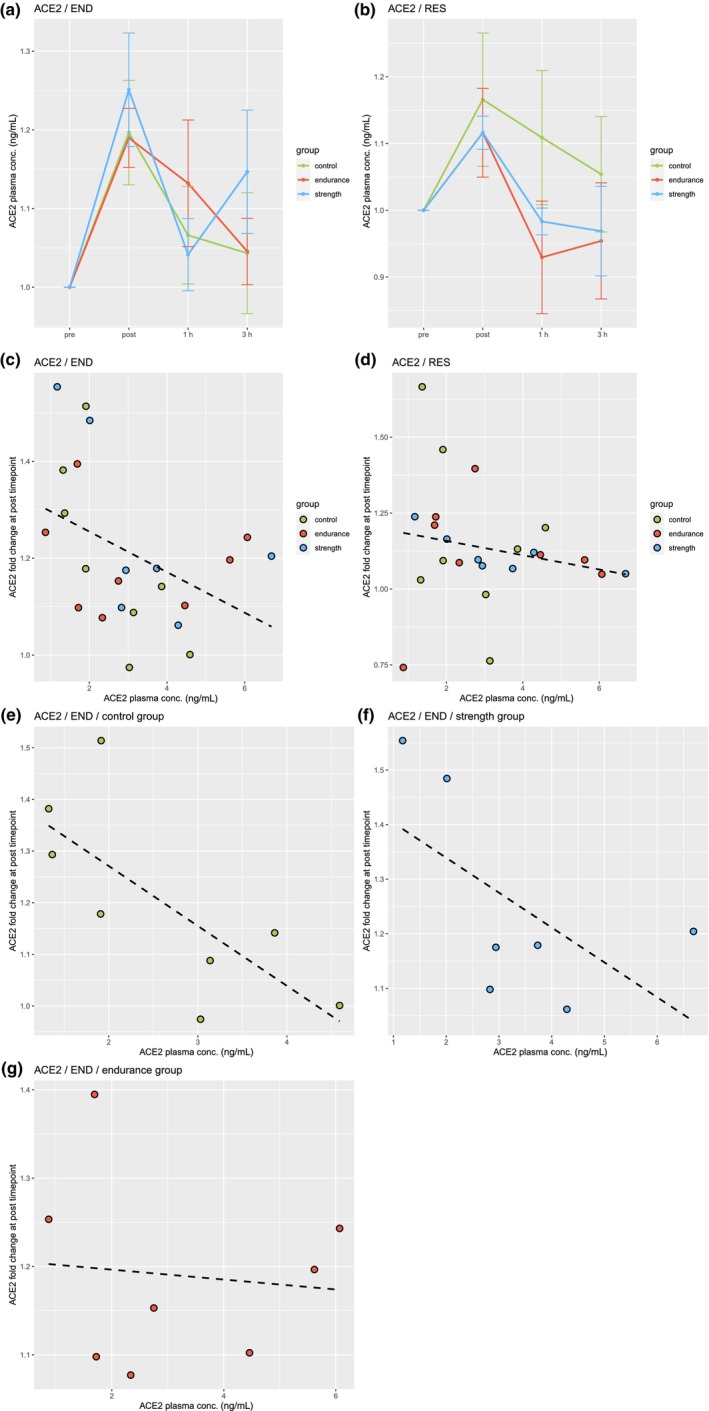
Acute response of circulating plasma ACE2 levels following acute endurance (END) and resistance exercise (RES). (a) All groups in response to END (±SE). (b) All groups in response to RES (±SE). (c) Correlation of plasma ACE2 fold change at post timepoint following END to baseline ACE2 plasma concentrations (*R* = −0.43). (d) Correlation of plasma ACE2 fold change at post timepoint following RES to baseline ACE2 plasma concentrations (*R* = −0.19). (e) Correlation of plasma ACE2 fold change at post timepoint following RES to baseline ACE2 plasma concentrations in control group (*R* = −0.74). (f) Correlation of plasma ACE2 fold change at post timepoint following RES to baseline ACE2 plasma concentrations in strength group (*R* = −0.6). (g) Correlation of plasma ACE2 fold change at post timepoint following RES to baseline ACE2 plasma concentrations in endurance group (*R* = −0.1).

In response to acute endurance exercise (END), ACE2 plasma levels within each subject immediately increased on average by 21%.On group level, this change was significant in CG, EG, and SG each immediately following the acute exercise (*p* < 0.05, Figure 2a). The extent of this change immediately following acute endurance exercise showed a strong negative correlation with ACE2 plasma levels at baseline (*R* = −0.43; Figure 2c). This showed to be particularly strong in CG and SG with a correlation of *R* = −0.74 and *R* = −0.6 respectively (Figure 2e,f), while EG showed no correlation (*R* = −0.1; Figure 2g).

In response to acute resistance exercise (RES), ACE2 plasma levels within each subject immediately increased on average by 13%. Here, only SG showed a significant increase (*p* < 0.05, Figure 2b). The change immediately following acute resistance exercise, unlike in response to END, did not strongly correlate with ACE2 baseline plasma levels (*R* = −0.19; Figure 2d). At the 1 h and 3 h timepoint, plasma ACE2 levels were not significantly different to baseline in any group or type of acute exercise intervention.

Subsequently, we performed a multiple regression analysis combining baseline ACE2 levels, BMI, and both VO_2_peak and leg strength results to analyze the contributions of different factors to the change in plasma ACE2 levels following acute exercise. Results show that in response to END, only baseline ACE2 levels significantly contribute to the model (*p* = 0.03), while BMI (*p* = 0.48), VO_2_peak (*p* = 0.82), and leg strength (*p* = 0.78) do not.

## 4 DISCUSSION

The main finding of this study was that baseline levels of circulating ACE2 were strongly negatively correlated with measures of fitness or health within each respective group. As a cleaver of the vasoconstrictor angiotensin II into the vasodilator angiotensin (1–7), membrane‐bound ACE2 contributes to the activation of the ACE2/angiotensin‐(1–7)/MAS axis, reducing systemic blood pressure (Santos et al., 2018). On the other hand, increased circulating ACE2 has been established as marker of risk of death and cardiometabolic disease (Narula et al., 2020). Thus, a lower level of circulating ACE2 can be seen as an indicator of improved cardiovascular health over‐all mortality. In lifelong trained high‐level endurance athletes, VO_2_peak can be used as a measure of fitness level (Butts et al., 1991), in lifelong trained high‐level strength athletes, muscle strength is representative of their fitness. In contrast, fitness of untrained healthy control subjects could be adequately separated by their BMI. Additionally, previous studies show that increased shedding of membrane‐bound ACE2 and thus increased amount of circulating ACE2 can be an early indicator of disease severity in SARS‐CoV‐2 during its onset (Mariappan et al., 2022). In the context of this shedding process, the strong negative correlation of baseline ACE2 levels with VO_2_peak in endurance athletes follows these previous findings. Similarly, in strength athletes, such a connection can be made with their skeletal muscle strength levels, here measured by leg strength. While VO_2_peak has been previously used to predict health complications such as cardiovascular and kidney diseases as well as general surgical outcome in lung diseases (Erman Helper et al., 2024; Gonzales et al., 2021; Salati & Brunelli, 2016), in the context of ACE2 neither VO_2_peak nor leg strength could be used to separate untrained control subjects. However, their BMI can, with the careful consideration of the implications of that metric such as body composition and height, be a relevant representation of their physical health. Such a correlation would not apply to the endurance and strength athletes, as their specific exercise training regimens would greatly distort such a relation, in strength athletes with exceptionally high BMI and in endurance athletes with a low BMI relative to their physical wellbeing, body composition and fitness (Etchison et al., 2011). The herein presented results would suggest a connection between fitness or health measures within each group and baseline ACE2 plasma concentration.

Interestingly, while ACE2 plasma levels somewhat increase with age across all groups, the degree of correlation between both varied with their training background. Age‐related differences in ACE2 plasma levels have been reported previously, with a positive correlation under and a negative correlation above the age of 55 (AlGhatrif et al., 2021). Interestingly, the endurance group showed the strongest correlation between age and ACE2 plasma levels. Furthermore, ACE2 levels were elevated particularly in response to acute endurance exercise, which is a more systemic form of exercise than acute resistance exercise, following which circulating ACE2 levels rose to a lesser extent. Some parts of this difference might potentially be explained by the hemoconcentration effect of endurance exercise (Alis et al., 2015). However, previous publications have investigated the different influence of acute endurance and resistance exercise on blood pressure and whole metabolic and immune system activation, concluding that endurance exercise exerts more extensive systemic vascular and circulatory system effects (Carpio‐Rivera et al., 2016; Schlagheck et al., 2020). Additionally, previous results from investigations of ACE2‐deficient mice might indicate that circulating ACE2 levels are positively correlated to endurance performance due to its vasodilatory effect (Motta‐Santos et al., 2016). While not significant, this might be in line with the higher average concentration of circulating ACE2 baseline in EG compared to both the other groups. Furthermore, despite correlating with baseline ACE2 levels, BMI, VO_2_peak and leg strength did not interact with the extent of ACE2 increase post‐exercise, while ACE2 baseline levels did. Together, this might provide an explanation for the stronger effect of acute endurance exercise on ACE2 plasma levels, and potentially the steeper ACE2‐age correlation in EG which might accumulate such ACE2‐affecting systemic effects that are consequences of their prolongating endurance training.

However, during the SARS‐CoV‐2 pandemic, endurance athletes showed to largely have a milder course and less complications compared to untrained patients (Krzywański et al., 2022). Soluble engineered ACE2 has further been successfully used to block COVID‐19 from entering cells by increasing its binding to increased circulating ACE2 levels instead of ACE2 bound to the plasma membrane, contributing to a reduced viral load (Monteil et al., 2020). An increase in circulating ACE2 following acute exercise might have a similar effect by binding COVID‐19 and blocking it from entering the cell. Despite having lower ACE2 baseline levels, potentially due to their repeated use of ACE2 shedding mechanisms, athletes might be more competent in activating such a cleavage process of membrane‐bound ACE2 when required in the context of both, acute exercise or a viral infection.

Taken together, in the context of previously published data about ACE2 and its mechanisms, the here presented finding of a strong negative correlation of plasma ACE2 levels with measures of cardiovascular fitness can add an interesting perspective to the dynamics of ACE2 with acute exercise and different training backgrounds that might help elucidate further the role of the dynamics of membrane‐bound and plasma‐circulating ACE2and contribute to our understanding of the health advantages of physical fitness.

## FUNDING INFORMATION

This study was supported by grants from Vetenskapsrådet (#2018‐02932) and Centrum för idrottsforskning (D2021‐0022; FO2022‐0005).

## CONFLICT OF INTEREST STATEMENT

The authors declare that they have no competing interests.

## ETHICS STATEMENT

The study was approved by the Stockholm regional ethics board (Dnr: 2016/590‐31) under observance of the declaration of Helsinki.

## Data Availability

The data that support the findings of this study are available from the corresponding author upon reasonable request.
